# Bane or Boon? The Role of Spirituality, Religion, and Well-Being in Later Life Across Diverse Older Populations

**DOI:** 10.1093/geroni/igaa057.2356

**Published:** 2020-12-16

**Authors:** Holly Nelson-Becker

## Abstract

Older adults tend to be religiously-affiliated to a greater extent than any other generational cohort (ARDA,2018; Koenig, King & Carson,2012; George et al.,2013; Nelson-Becker,2018). However, their level of engagement varies across cultural and national contexts. Complex life course trajectories related to spirituality and religion mean that these domains often interface with both challenges and a search for well-being. Individuals may align with spiritual and/or religious values to a greater or lesser extent at different periods in their lives becoming more spiritual/religious, less spiritual/religious, or differently so. These papers address diverse perspectives on spirituality, religion, and well-being using samples primarily from the UK, Europe, the US, and Canada. The first paper by Christina Victor sets context by comparing the role of religion, and spirituality in well-being across three separate older adult data sets, touching on loneliness and dementia. Holly Nelson-Becker discusses results from an online international survey of older persons who walked the ancient Camino de Santiago pilgrimage regarding their motivations and learning from the experience. Michael Thomas considers the complex role of spirituality and sexuality for older LGB couples who may choose to remain in or leave their religious faith as they integrate expanding views. Keith Anderson explores perceptions of belief changes among religious and spiritual older adults across the life course. Together, these papers will address benefit and harm from formal religious practice and the advancing roles of spirituality as well as nonspirituality (the “nones”) in global societies.

